# Identification of a novel AP2 transcription factor in zygotes with an essential role in *Plasmodium* ookinete development

**DOI:** 10.1371/journal.ppat.1010510

**Published:** 2022-08-10

**Authors:** Tsubasa Nishi, Izumi Kaneko, Shiroh Iwanaga, Masao Yuda

**Affiliations:** 1 Laboratory of Medical Zoology, Department of Medicine, Mie University; 2 Department of Molecular Protozoology, Research Institute for Microbial Diseases, Osaka University; Francis Crick Institute, UNITED KINGDOM

## Abstract

The sexual phase of *Plasmodium* represents a crucial step in malaria transmission, during which these parasites fertilize and form ookinetes to infect mosquitoes. *Plasmodium* development after fertilization is thought to proceed with female-stored mRNAs until the formation of a retort-form ookinete; thus, transcriptional activity in zygotes has previously been considered quiescent. In this study, we reveal the essential role of transcriptional activity in zygotes by investigating the function of a newly identified AP2 transcription factor, AP2-Z, in *P*. *berghei*. *ap2-z* was previously reported as a female transcriptional regulator gene whose disruption resulted in developmental arrest at the retort stage of ookinetes. In this study, although *ap2-z* was transcribed in females, we show that it was translationally repressed by the DOZI complex and translated after fertilization with peak expression at the zygote stage. ChIP-seq analysis of AP2-Z shows that it binds on specific DNA motifs, targeting the majority of genes known as an essential component of ookinetes, which largely overlap with the AP2-O targets, as well as genes that are unique among the targets of other sexual transcription factors. The results of this study also indicate the existence of a cascade of transcription factors, beginning with AP2-G, that proceeds from gametocytogenesis to ookinete formation.

## Author summary

Sexual development in *Plasmodium* parasites, a causative agent of malaria, is essential for their transmission from vertebrate hosts to mosquitoes. This important developmental process proceeds as follows: formation of a gametocyte/gamete, fertilization and conversion of the zygote into the mosquito midgut invasive stage, called the ookinete. As a target of transmission blocking strategies, it is important to understand the mechanisms regulating *Plasmodium* sexual development. In this study, we assessed transcriptional regulation after fertilization by investigating the function of a novel transcription factor, AP2-Z. The results revealed the essential role of *de novo* transcription activated by AP2-Z in zygotes for promoting ookinete development. As transcriptional activity during the zygote stage has previously been considered silent in *Plasmodium*, novel genes important for ookinete formation can now be explored in the target genes of AP2-Z. Investigating the functions of these genes can help us understand the mechanisms of *Plasmodium* zygote/ookinete development and identify new targets for transmission blocking vaccines.

## Introduction

Malaria is a serious infectious disease caused by *Plasmodium* parasites of the Apicomplexa phylum, which are propagated among humans through bites from Anopheles mosquitoes [1]. Successful transmission from vertebrate hosts to mosquitoes requires these parasites to proceed with complex sexual development [2,3]; therefore, understanding the mechanism of *Plasmodium* sexual development is a crucial aspect of malaria epidemiology.

*Plasmodium* sexual development begins when a subpopulation of asexual blood-stage parasites differentiate into male and female gametocytes in the host blood stream [4,5]. Taken up through mosquito blood feeding, these gametocytes then develop into gametes and fertilize. The fertilized female gametes, or zygotes, then develop into a banana-shaped motile stage known as ookinetes, which then invade the midgut epithelia to proceed with further development beneath the basal lamina [6].

*Plasmodium* sexual development is regulated at both transcriptional and translational levels. At the beginning of sexual development, gametocytogenesis is induced by the AP2 transcription factor AP2-G, which is expressed in a subpopulation of blood-stage parasites [7–10]. Subsequently, the transcriptional repressor AP2-G2 starts to be expressed in the early stage and supports differentiation to gametocytes [11,12]. In addition, AP2-G5, another transcriptional repressor, promotes development of early gametocytes [13]. Early gametocytes then differentiate into male and female gametocytes. For females, two transcription factors, AP2-FG and AP2-O3, function as a transcriptional activator and repressor, respectively, to promote development of female gametocytes [14,15]. In addition, during female development, parasites prepare for development after fertilization by preserving mRNAs [16]. This preservation of mRNAs is conducted by a translational repressor complex, in which at least two factors, ATP-dependent RNA helicase DDX6 (DOZI) and trailer hitch homolog (CITH), are currently known to be involved [16–19].

In *Plasmodium berghei*, fertilized females, called zygote, undergo meiosis within 4 h post-fertilization [18]. After 6–8 h of zygote development, parasites start forming an apical protrusion, becoming retort-form ookinetes, and then develop into mature ookinetes approximately 20 h post-fertilization [20]. For these processes, female-stored mRNA plays an essential role; parasites that lack *dozi* are not able to begin either meiosis or apical protrusion formation [16,18]. In contrast, the role of *de novo* transcripts in zygotes has not been investigated, although it has been shown that *Plasmodium* zygotes can develop into retort-form ookinetes even in the presence of a transcriptional inhibitor [19]. Therefore, during the development of *Plasmodium* zygotes, transcriptional activity is considered to be quiescent, similar to animal embryos whose transcriptional activity is quiescent during the early stage after fertilization [21,22]. For the late stage of ookinete development, parasites must activate *de novo* transcription. AP2-O is one transcription factor that is expressed from retort-form ookinetes to mature ookinetes [23], which activates the majority of known ookinete genes; thus, disruption of *ap2-o* results in impaired development of ookinetes [24]. In addition, AP2-O2 is also essential for ookinete maturation in *P*. *berghei* [25], although disruption of *ap2-o2* in *P*. *yoelii* does not affect ookinete development [26]. Thus, according to existing studies of transcriptional and translational regulators, *Plasmodium* ookinete development is currently considered to be promoted by female-stored mRNAs in the early stage and *de novo* transcripts in the late stage.

In a previous study, we identified an AP2 transcription factor-related gene, *ap2r-1*, as a target gene of AP2-G and AP2-FG, and showed that its disruption affects ookinete development [10]. Here, we demonstrate that AP2R-1 is an AP2 transcription factor that functions in zygotes; hence, we rename it AP2-Z. By investigating the functions of AP2-Z, we reveal the essential role of *de novo* transcription in zygotes for ookinete formation. Moreover, we also demonstrate the existence of a transcriptional cascade, which starts with AP2-G, during *Plasmodium* development from gametocytes to ookinetes.

## Results

### *ap2-z* is an AP2-family protein gene conserved in Apicomplexan parasites

**Fig 1:**
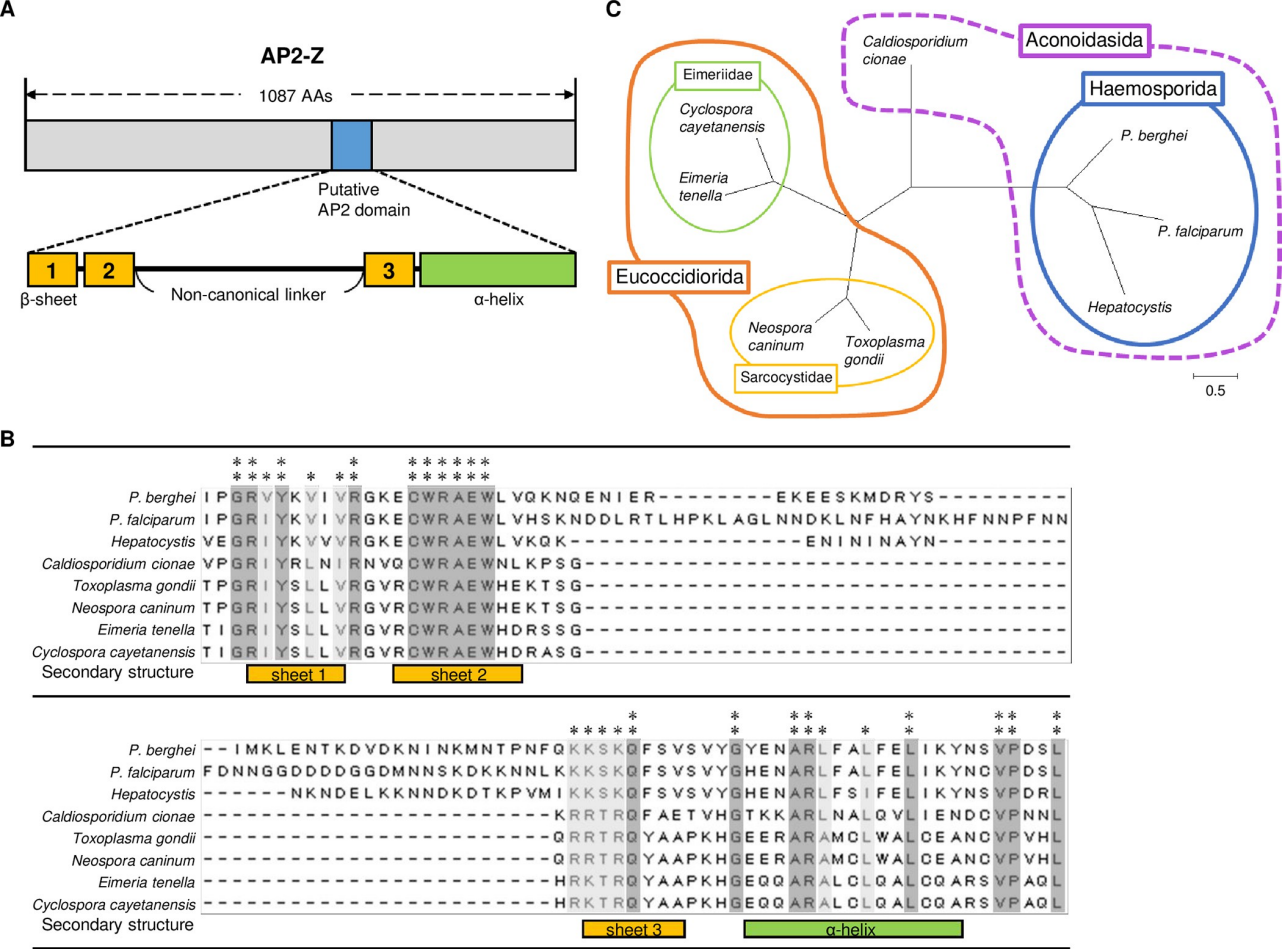
Identification of possible AP2-Z orthologs in Apicomplexan parasites. (A) A schematic illustration of AP2-Z. Blue box shows the AP2 domain. Beta-sheet and α-helix regions in the AP2 domain were predicted using PSIPRED (http://bioinf.cs.ucl.ac.uk/psipred/) and indicated in orange and green boxes, respectively. (B) Alignment of conserved amino acid sequences from AP2-Z and blastp-searched proteins by the ClustalW program in Mega X. Positions at which all sequences have an identical amino acid are indicated by two asterisks, whereas positions with amino acid residues of the same property are indicated by one asterisk. Amino acid sequences were retrieved from PlasmoDB or the NCBI database (*P*. *berghei*, PBANKA_0612400; *P*. *falciparum*, PF3D7_0411000; *Hepatocystis*, VWU49277; *Cardiosporidium cionae*, KAF8822898; *Toxoplasma gondii*, XP_018635762; *Neospora caninum*, XP_003884720; *Eimeria tenella*, XP_013229162; *Cyclospora cayetanensis*, OEH74626). (C) Phylogenetic tree of AP2-Z and blastp-searched proteins, which was inferred from their whole amino acid sequences using the Maximum Likelihood method and JTT matrix-based model. Tree is drawn to scale, with branch lengths measured according to the number of substitutions per site. Species of the same family, order, and class are enclosed by thin, thick, and dashed lines, respectively.

We previously identified *ap2-z* (PBANKA_0612400) as a target gene of AP2-G and AP2-FG. This gene is expressed in female gametocytes, and its transcription is highly female-specific in both *P*. *berghei* and *P*. *falciparum* (approximately 100-fold and 20-fold higher in female gametocytes compared to male gametocytes, respectively) as shown by studies that performed stage-specific RNA-seq analysis [27–29]. Disruption of *ap2-z* results in arrested zygote development at the retort stage of ookinetes although meiotic replication was not affected as we assessed by fluorescence-activated cell sorting (FACS) analysis (S1 Fig). AP2-Z has a putative AP2 domain that has not been identified by homology searches because of a non-canonical linker peptide between the second and third sheet (Fig 1A). The domain is highly conserved in the *Plasmodium* species except for the linker peptide, which varies in amino acid sequence and length among the species (S2 Fig). To investigate whether AP2-Z is conserved in other organisms, we searched for any proteins with a domain similar to the putative AP2 domain of AP2-Z. Protein–protein BLAST (blastp) search identified proteins with a homologous domain to the putative AP2 domain of AP2-Z from Apicomplexan parasites, such as *Toxoplasma gondii* and *Eimeria tenella* (Fig 1B). The conserved amino acids among *Plasmodium* AP2-Z and these blastp-searched proteins were mostly found within the putative beta sheets and alpha helix, and did not have the non-canonical linker peptide, except for the protein from *Hepatocystis* (Fig 1B). Notably, these conserved amino acids contained consensus amino acids for the AP2 domain [30], indicating that this conserved domain is actually an AP2 domain. The homologous protein from *Toxoplasma* is consistently annotated as “AP2 domain transcription factor AP2X-6,” and the homologous domain from all blastp-searched proteins without the non-canonical linker was assigned as “AP2” in a protein domain search using a Simple Modular Architecture Research Tool (SMART, http://smart.embl-heidelberg.de/). The phylogenetic tree of *Plasmodium* AP2-Z and the blastp-searched proteins was topologically consistent with the species tree of Apicomplexa, which further suggested that these proteins are orthologs (Fig 1C). In this regard, their ancestral protein probably did not include the non-canonical linker peptide within the conserved domain, with *Plasmodium* and *Hepatocystis* species acquiring this linker during evolution instead. Collectively, we concluded that AP2-Z is an AP2-family protein conserved in the phylum Apicomplexa.

### *ap2-z* is translationally repressed in female gametocytes by the DOZI complex and expressed in zygotes

**Fig 2:**
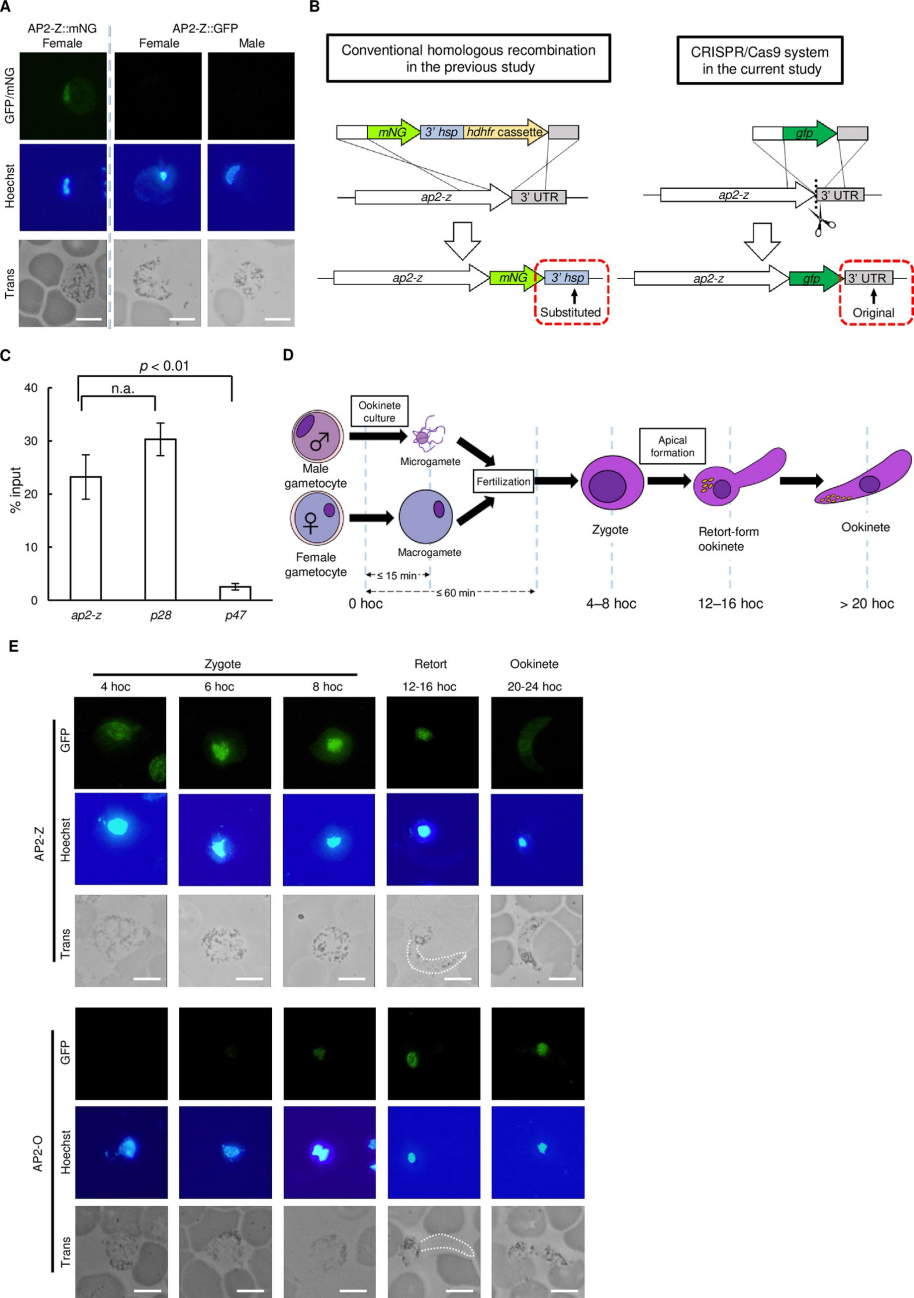
Expression profile of AP2-Z during sexual development and its translational repression in females. (A) Expression of AP2-Z in gametocytes of the AP2-Z::mNG (left) and the AP2-Z::GFP parasite (right). Nuclei were stained with Hoechst 33342. Scale bar = 5 μm. (B) Schematic illustrations of fluorescent protein-tagging strategies of AP2-Z. In the AP2-Z::mNG parasite, the 3’ UTR of *ap2-z* was substituted upon fusion with *mNG* (left). In the AP2-Z::GFP parasite, the endogenous 3’UTR was retained upon *gfp*-fusion performed by CRISPR/Cas9 (right). (C) Enrichment of DOZI-associated transcripts evaluated by RNA immunoprecipitation-qPCR analysis. Pre-immunoprecipitated samples were used as the input. Enrichment of *p28* and *p47* transcripts was analyzed as a positive and negative control, respectively. The *p*-value on the graph was calculated by Student’s t-test. Error bars indicate the standard error of the mean (n = 3). (D) Schematic illustration of zygote/ookinete development in ookinete cultures for *P*. *berghei*. When gametocytes are subjected to ookinete culture medium, gamete activation is induced by low temperature and rise in pH, which completes within 15 min. The gametes then find a mate and complete fertilization process, including cell fusion and nuclear fusion, within 60 min after starting ookinete cultures. After fertilization, zygote development takes for 6–8 h, and then the zygotes become retort-form ookinetes starting formation of apical protrusion. Finally, the parasites develop into mature ookinetes in approximately 20 hoc. (E) Expression of AP2-Z and AP2-O during ookinete culture using the AP2-Z::GFP parasite and the parasite expressing GFP-fused AP2-O, which generated in the previous study [23], respectively. Nuclei were stained with Hoechst 33342. Apical protrusion of the retort-form ookinete at 16 hoc is highlighted with a white dashed line. Scale bar = 5 μm.

In our previous study, we tagged AP2-Z with mNeonGreen fluorescent protein (mNG) using a conventional homologous recombination method (AP2-Z::mNG), and observed a fluorescent signal in the nucleus of female gametocytes (Fig 2A), consistent with the previous observation that it is a target of AP2-G and AP2-FG [10]. To evaluate whether *ap2-z* is actually activated by AP2-G and AP2-FG, we planned to perform a promoter assay on the endogenous *ap2-z*: assessing roles of binding motifs of these two transcription factors as a *cis*-regulatory element by introducing mutations on them. However, when we developed parasites expressing green fluorescent protein (GFP)-fused AP2-Z (AP2-Z::GFP) by the CRISPR/Cas9 system using the Cas9-expressing parasite (PbCas9) [31] (S3A Fig) to perform the promoter assay, we observed no fluorescent signal in any blood-stage parasites, including female gametocytes (Fig 2A).

In the homologous recombination method used previously, the 3’ untranslated region (UTR) of *ap2-z* was replaced by that of *hsp70* upon tagging AP2-Z with mNG (Fig 2B, left) [10]. However, in this study, we inserted *gfp* at the 3’ end of *ap2-z* by CRISPR/Cas9 without replacing the original 3’ UTR (Fig 2B, right). In female gametocytes of the *Plasmodium* species, a subset of genes is translationally repressed by the DOZI complex in a 5’ UTR- or 3’ UTR-dependent manner [17,19]. Therefore, we assumed that *ap2-z* is translationally repressed by the DOZI complex in normal females but translated in females of AP2-Z::mNG because of the replacement of 3’ UTR. Consistent with this assumption, strong fluorescence was observed in the nucleus of AP2-Z::GFP zygotes.

To further address whether *ap2-z* is translationally repressed by the DOZI complex, we developed parasites expressing GFP-fused DOZI (DOZI::GFP) and performed RNA immunoprecipitation (RIP) followed by reverse transcription quantitative polymerase chain reaction (RT-qPCR) analysis. The analysis showed that more than 20% of *ap2-z* transcript was immunoprecipitated with anti-GFP antibody (Fig 2C). The percentage was comparable to that of *p28*, which is known as a target of the DOZI complex [16,19]. On the other hand, the transcript of *p47*, which is a gene translated in females [32], was only slightly detected in the immunoprecipitated samples. Therefore, these results validated that *ap2-z* was translationally repressed by the DOZI complex in female gametocytes.

Given the above results, we next assessed the expression of AP2-Z in a time course manner during zygote/ookinete development using the AP2-Z::GFP parasite. We performed ookinete culture in which parasite development proceeds generally as illustrated in Fig 2D. In ookinete cultures, gamete activation occurs within 15 min, and fertilization within 60 min. After fertilization, zygote development continues until approximately 8 h after the start of ookinete culture (hoc), and then parasites start forming apical protrusion. Most fertilized parasites develop into mature ookinetes in approximately 20 hoc. Nuclear-localized fluorescence began to appear in zygotes at 4 hoc and became stronger at 6 hoc (Fig 2E). The strong fluorescence continued until zygotes started forming an apical protrusion (8–12 hoc). The signal then faded in retort-form ookinetes and became barely detectable in the nuclei of banana-shaped ookinetes (Fig 2E). Therefore, AP2-Z is mainly expressed in zygotes prior to apical protrusion formation. Considering the expression of AP2-O in the late stage of ookinete development (Fig 2E) [23], these results might indicate that AP2-Z is responsible for transcriptional regulation until AP2-O starts to be expressed.

### *ap2-z* is activated by AP2-G and AP2-FG in female gametocytes

**Fig 3:**
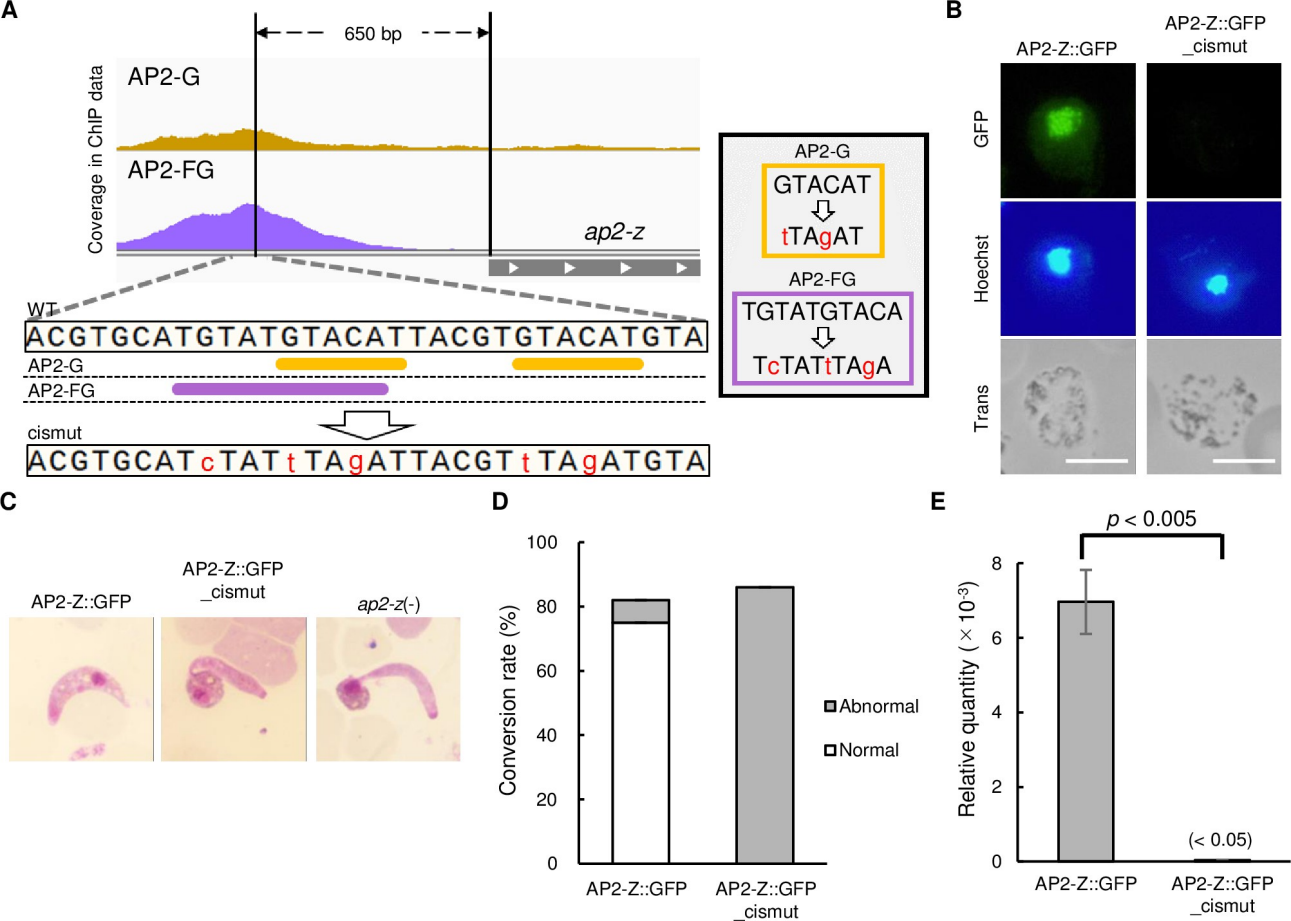
Transcriptional activation of *ap2-z* by AP2-G and AP2-FG. (A) Integrative Genomics Viewer images from the ChIP-seq data of AP2-G and AP2-FG for the upstream region of *ap2-z*. Binding motifs of AP2-G and AP2-FG in the peak region are indicated by orange and purple bars, respectively. Mutations introduced to these motifs are described on the right. The same mutations were introduced to both of the two AP2-G motifs. (B) Expression of AP2-Z in the AP2-Z::GFP_cismut at 6 hoc. Nuclei were stained with Hoechst 33342. Scale bar = 5 μm. (C) Representative Giemsa-stained image of ookinete in AP2-Z::GFP, AP2-Z::GFP_cismut, and *ap2-z*(-) parasites at 20 hoc. (D) Rate of conversion to ookinetes against all female-derived cells in AP2-Z::GFP and AP2-Z::GFP_cismut at 20 hoc. (E) Relative amounts of *ap2-z* transcripts in gametocyte assessed by RT-qPCR analysis. The *p28* gene was used as the internal control. Value for AP2-Z::GFP_cismut is given in brackets as it is too small to be depicted in the graph. The *p*-value on the graph was calculated by the Student’s t-test. Error bars indicate the standard error of the mean (n = 3).

To evaluate whether *ap2-z* transcription is activated downstream of AP2-G and AP2-FG, we disrupted their binding motifs located upstream of *ap2-z* by CRISPR/Cas9 using AP2-Z::GFP parasites (AP2-Z::GFP_cismut) (Figs 3A and S2B) and assessed the expression of AP2-Z in the zygotes. The upstream region of *ap2-z* has two binding motifs of AP2-G [10] and one motif of AP2-FG [14]. These motifs are located near to each other, with one of the AP2-G motifs overlapping with the AP2-FG motif, at approximately 650 bp upstream from the start codon of *ap2-z* (Fig 3A). Disruption of these three motifs resulted in complete loss of fluorescence in the nucleus of zygotes (Fig 3B). Furthermore, the AP2-Z::GFP_cismut parasites lost the ability to produce banana-shaped ookinetes, as with *ap2-z* knockout parasites [*ap2-z*(-)] (Fig 3C and 3D). We further examined the expression of *ap2-z* in the AP2-Z::GFP_cismut at the RNA level. Mice infected with AP2-Z::GFP or AP2-Z::GFP_cismut parasites were treated with sulfadiazine to enrich gametocytes by killing asexual blood-stage parasites. Total RNA was then harvested from the infected blood and subjected to RT-qPCR analysis. The amount of *ap2-z* transcripts relative to that of *p28* was downregulated more than 200-fold in AP2-Z::GFP_cismut compared to that in AP2-Z::GFP (Fig 3E). Collectively, these results suggested that transcription of *ap2-z* is activated by AP2-G and AP2-FG in the early and female gametocyte, respectively.

### AP2-Z binds to specific sites of the genome recognizing (T/C)(A/C)TG(A/T)AC(A/G) motifs

**Fig 4:**
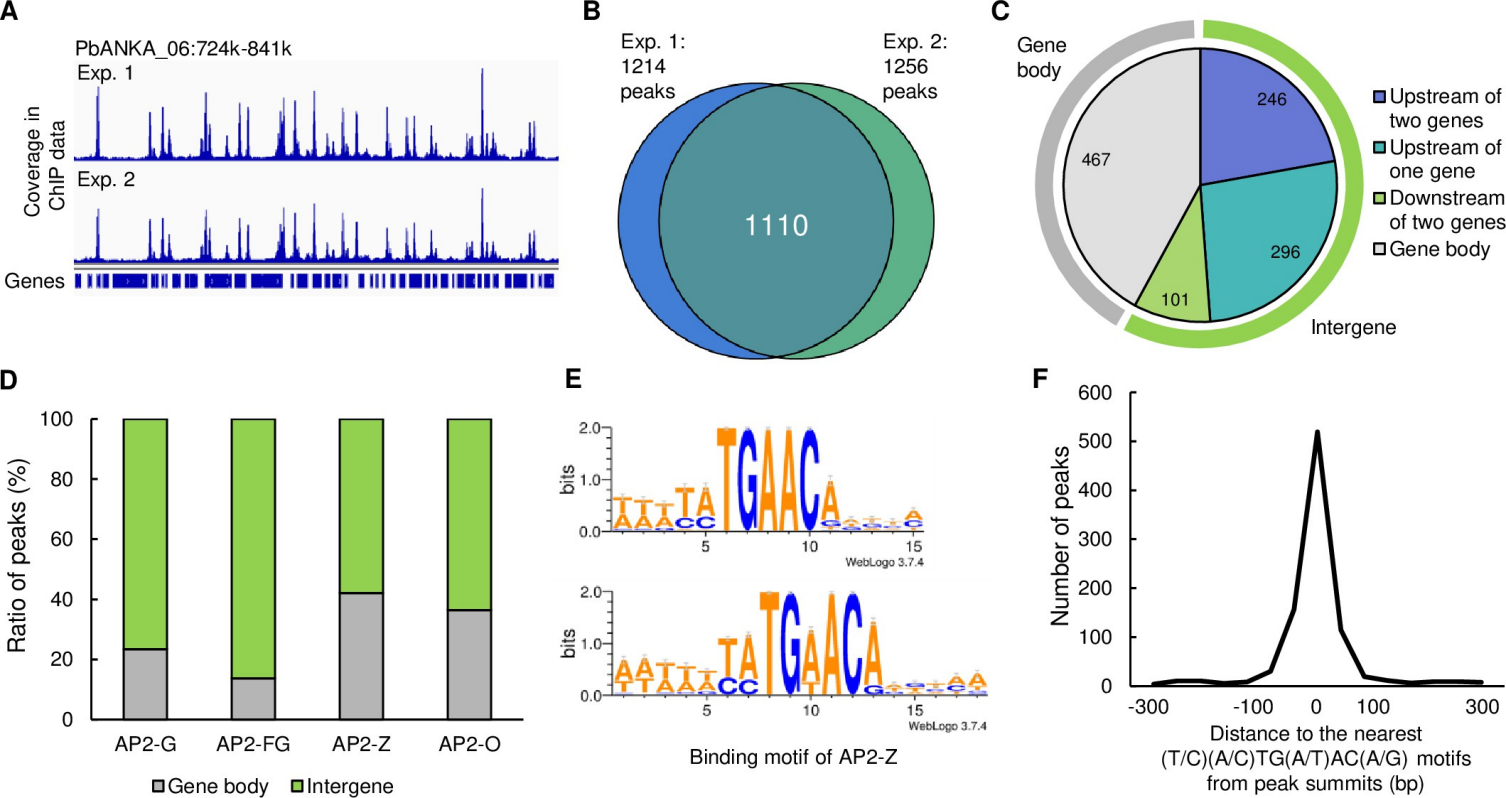
ChIP-seq analysis of AP2-Z. (A) Integrative Genomics Viewer images from ChIP-seq experiment 1 and 2 of AP2-Z in a part of chromosome 6. Histograms show sequence coverage of ChIP data at each base. (B) Venn diagram of number of peaks identified in ChIP-seq experiment 1 and 2. (C) Classification of peak locations. Light green and gray bars around the pie chart indicate the intergenic region and gene body region, respectively. (D) Ratio of peaks located on the gene body and intergenic region for ChIP-seq of AP2-G, AP2-FG, AP2-Z, and AP2-O. (E) Sequence logo constructed from TGAAC motifs in the peak regions (top) and predicted binding motif of AP2-Z (bottom). Sequence logos are depicted using WebLogo 3 (http://weblogo.threeplusone.com/create.cgi). (F) Histogram showing the distance between the peak summit and the nearest (T/C)(A/C)TG(A/T)AC(A/G) motif for each ChIP peak.

To assess the target genes of AP2-Z, we performed chromatin immunoprecipitation followed by high-throughput sequencing (ChIP-seq) analysis using AP2-Z::GFP parasites. The parasites were harvested at 6 hoc, when parasites have not yet developed into retort-form or mature ookinete, and subjected to ChIP-seq experiments using anti-GFP antibody, and the experiments were performed in duplicate. The overall peak patterns were similar between each replicate, indicating good reproducibility of the two experiments (Fig 4A). Experiment 1 and 2 identified 1,214 and 1,256 peaks with fold enrichment > 4.0, respectively, with 1,110 overlapping peaks between the two experiments (Fig 4B, S1A and S1B Table).

Of the 1,110 common peaks, 643 peaks (57.9%) were located within intergenic regions, most of which were located upstream of the genes (Fig 4C). The ratio of peaks located on gene bodies (42.1%) was clearly higher than that in the ChIP-seq of AP2-G and AP2-FG, which were expressed in the gametocyte stages (Fig 4D) [10,14]. However, when we analyzed our previous ChIP-seq data of AP2-O, which is an essential transcription factor for ookinete development, the ratio of peaks located on gene bodies was 36.5%, which was comparable to that of AP2-Z (Fig 4D) [24].

Next, we searched for motifs enriched in the peak regions to predict the binding motif of AP2-Z. Motif enrichment analysis by Fisher’s exact test showed that the TATGAACA motif was most enriched within 50 bp from the peak summits, with a *p*-value of 3.69 × 10^−215^. We then identified enrichment of several further motifs that contained TGAAC and, taking them together, obtained the (T/C)(A/C)TGAAC(A/G) motif. When constructing the sequence logo by WebLogo3 using the sequences around the TGAAC motifs nearest to each peak summit, the above 8-bp motif was consistently depicted (Fig 4E, top). In addition to the (T/C)(A/C)TGAAC(A/G) motif, we also found enrichment of motifs that match (T/C)(A/C)TGTACA with a *p*-value of 5.16 × 10^−84^. Taken together, we obtained the (T/C)(A/C)TG(A/T)AC(A/G) motif as a putative binding motif of AP2-Z (Fig 4E, bottom). The motif was identified within 100 bp from the summits of 75.5% peaks; the closer the motif was to the summits, the more peaks were identified (Fig 4F).

### AP2-Z broadly targets genes that are important for ookinete development

**Fig 5:**
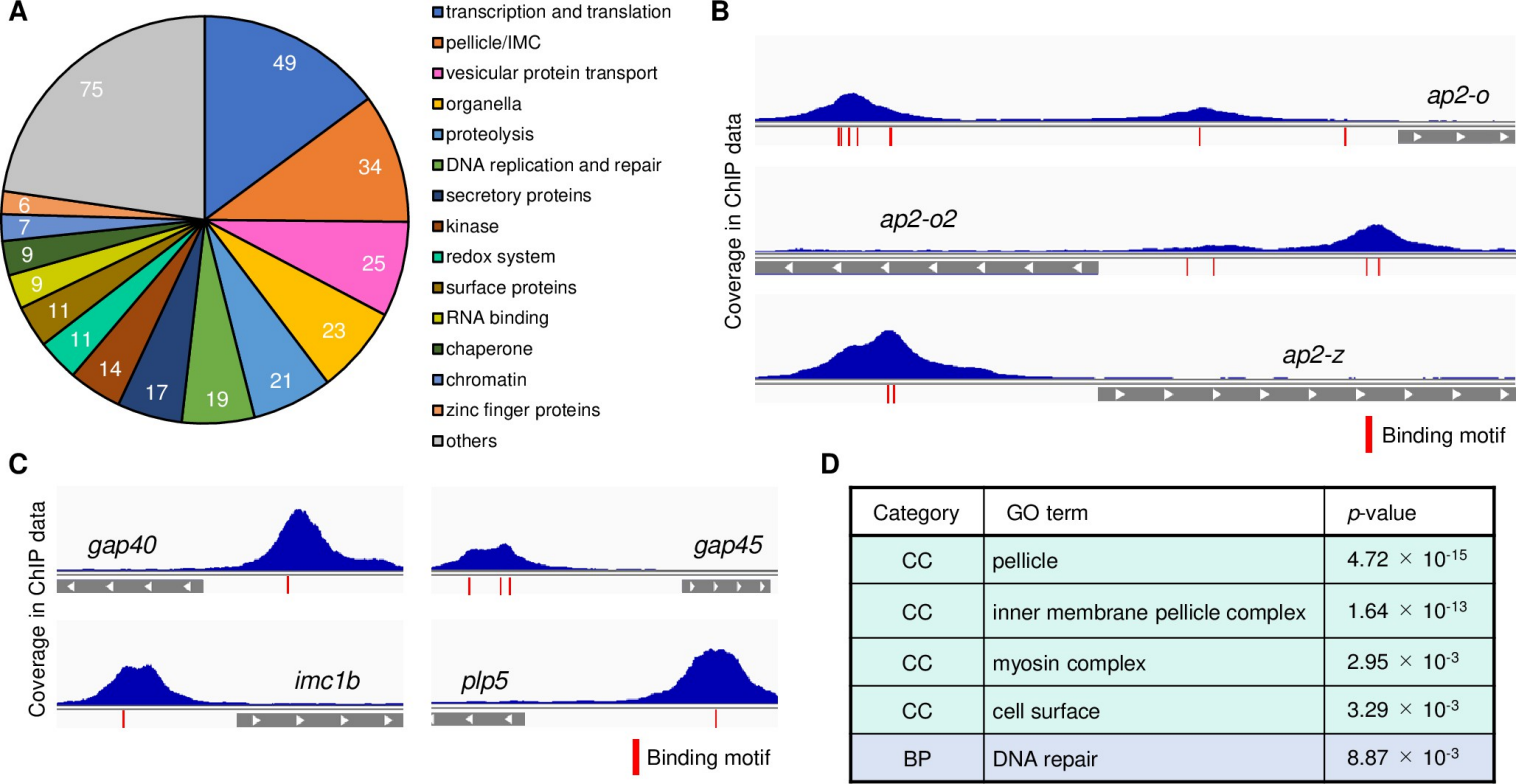
Target analysis of AP2-Z using ChIP-seq data. (A) Classification of the target genes of AP2-Z into 14 functional groups according to their product description annotated on PlasmoDB. (B) Transcription factor genes in the targets of AP2-Z. Histograms show sequence coverage of the ChIP sample in experiment 1. Gray bars indicate a partial open reading frame. Positions of the AP2-Z binding motifs are indicated in red. (C) Representative peak images of target genes essential for ookinete development. (D) GO analysis showing enrichment of genes with specific functions in the targets of AP2-Z. Terms with *p*-value < 0.01 were shown. CC = Cellular Components, BP = Biological Process.

Next, we identified the target genes of AP2-Z from the ChIP-seq data. The analysis showed that AP2-Z was bound within the 1,200-bp region upstream of 516 genes (S2 Table). To evaluate the role of AP2-Z in zygote development, we classified these target genes into several groups according to their product descriptions annotated on PlasmoDB (https://plasmodb.org/plasmo/app/). The target genes contained 330 functionally annotated genes. Of these annotated target genes, 49 genes were categorized to the group ‘transcription and translation,’ which was the largest of 14 functional groups (Fig 5A). This group contained putative translation initiation factor genes, such as *eIF3b* and *eIF6*, elongation factor genes, and genes related to ribosomal biogenesis. Furthermore, the group ‘transcription and translation’ also included *ap2-o*, which is the gene coding a transcription factor essential for ookinete maturation (Fig 5B). In addition, although not identified as a target because of the 1,200-bp cutoff, we found a peak at approximately 1,500 bp upstream of *ap2-o2*. Considering the fact that *ap2-z* is activated by AP2-G and AP2-FG, these results suggested the existence of a cascade of transcription factors, starting from AP2-G, during the sexual development of *Plasmodium*. Besides *ap2-o* and *ap2-o2*, *ap2-z* itself was also included in the targets, suggesting the existence of transcriptional autoregulation (Fig 5B), a characteristic widely observed during prokaryotic and eukaryotic transcriptional regulation [33,34]. As AP2-G and AP2-FG are no longer expressed after fertilization, this autoregulation is probably required for the stable expression of AP2-Z during zygote development.

Other than the ‘transcription and translation’ group, the target genes largely contained genes that are important for ookinete development (Fig 5C). The group ‘pellicle/IMC’ included *gap40*, *gap45*, and 15 inner membrane complex (IMC) protein genes, which are related to the pellicle structure of ookinetes. Most of the known ookinete microneme genes, such as *plp3*, *celtos*, and *ctrp*, were included in the group ‘secretory proteins.’ Gene ontology (GO) analysis further supported these results. In the category ‘Cellular Components (CC)’, the target genes of AP2-Z were most enriched in ‘pellicle,’ with a *p*-value of 4.72 × 10^−15^ (Fig 5D). Furthermore, in the category ‘Biological Process (BP)’, the target genes were most enriched in ‘DNA repair,’ which indicated that AP2-Z may also contribute to genome stability after meiotic replication in the zygote stage.

### Target genes of AP2-Z are activated in early zygotes

**Fig 6:**
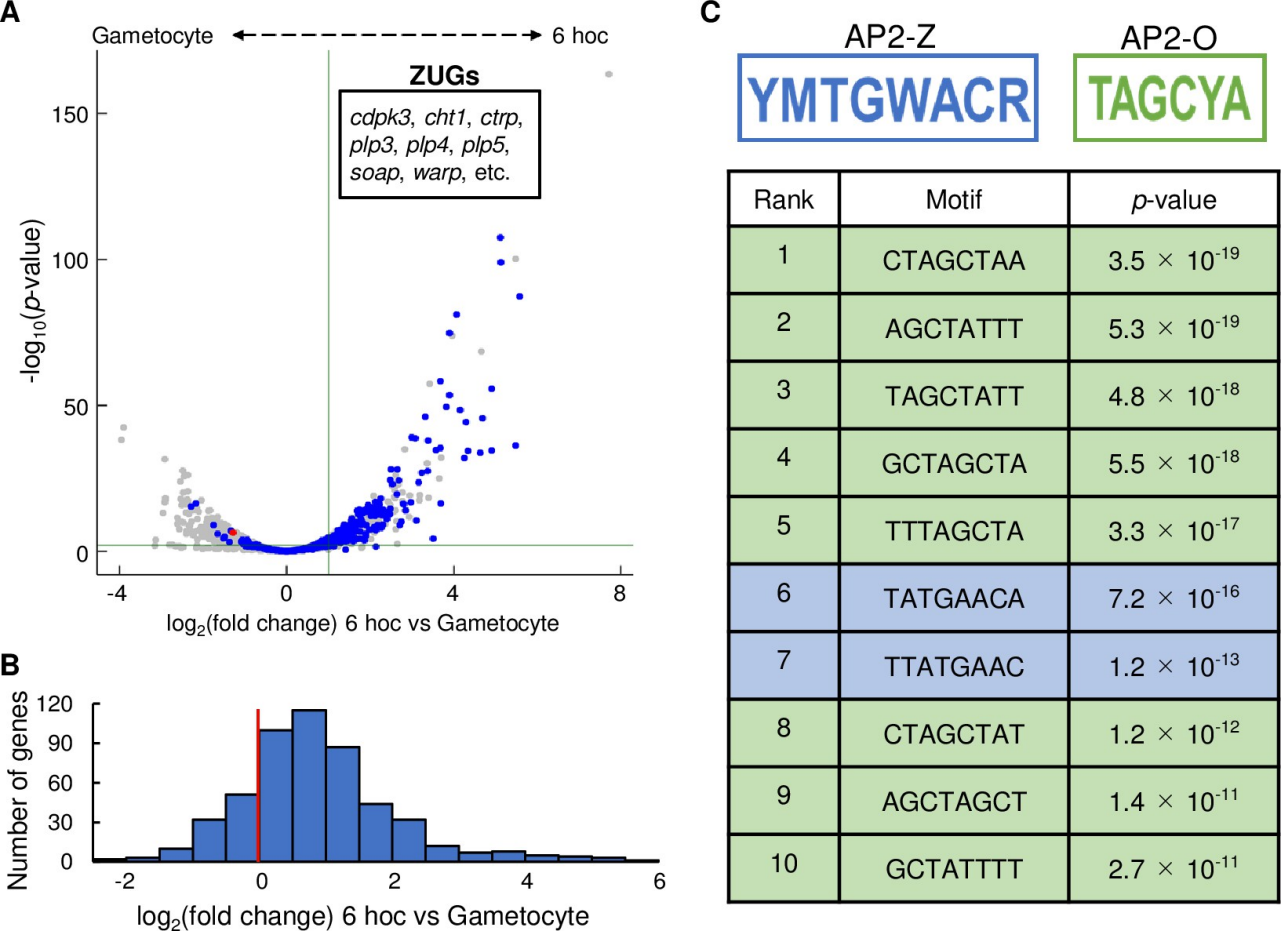
Differential expression analysis between gametocytes and zygotes at 6 hoc. (A) Volcano plot showing differential expression analysis between gametocytes and zygotes at 6 hoc. Red dot represents *ap2-z* and blue dots represent the target genes of AP2-Z. Green horizontal line indicates a *p*-value of 0.05 and a vertical line indicates a log_2_(fold change) of 1. (B) Histogram of the number of AP2-Z targets against the log_2_(fold change) value (bin = 0.5) in the differential expression analysis. Red vertical line indicates a log_2_(fold change) of 0. (C) Eight-base motifs enriched in the upstream of zygote-upregulated genes. AP2-Z motifs are indicated in blue and AP2-O motifs are indicated in green. Motifs were ranked according to their *p*-values.

Since *de novo* transcription in zygotes were previously considered silent, we next investigated whether the target genes of AP2-Z are actually transcribed in the zygotes. We performed RNA-seq analysis on the wild-type *Plasmodium berghei* ANKA strain (WT) at the gametocyte stage and 6 hoc to identify genes upregulated after fertilization. Compared to gametocytes, 512 genes were significantly upregulated (log_2_(fold change) > 1, *p*-value adjusted for multiple testing according to the Benjamini–Hochberg procedure (*p*-value adj) < 0.01) in the parasites at 6 hoc (Fig 6A, S3 Table). These zygote-upregulated genes (ZUG) contained known ookinete genes, such as *cdpk3*, *celtos*, and *ctrp*, indicating that transcription of these ookinete genes already occurs during the early stage of zygote development. Of the 512 ZUGs, 198 genes were targets of AP2-Z, showing significant enrichment with a *p*-value of 3.7 × 10^−69^ by Fisher’s exact test. Furthermore, more than 80% of the target genes were more highly expressed in zygotes at 6 hoc than in gametocytes (log_2_(fold change) > 0) (Fig 6B), indicating that the targets of AP2-Z were transcribed after fertilization. Detecting significant upregulation in only a subset of the target genes (38% of the targets) was probably due to the overlap with female-stored mRNAs. Collectively, these results indicated that the target genes of AP2-Z are actively transcribed during the zygote stage.

To further confirm the relationship between the target genes of AP2-Z and the ZUGs, we investigated whether the binding motif of AP2-Z was enriched in the upstream region of the ZUGs. We assessed enrichment of any motif in the upstream region (300–1,200 bp from the start codon) of ZUGs compared to that of the other genes by Fisher’s exact test. In the upstream region of ZUGs, TATGAACA, one of the binding motifs of AP2-Z [(T/C)(A/C)TG(A/T)AC(A/G)] was enriched, with a *p*-value of 7.2 × 10^−16^ (Fig 6C). Furthermore, another motif that partially contained the binding motif of AP2-Z, TTATGAAC, was also significantly enriched, with a *p*-value of 1.2 × 10^−13^. This result strongly indicated that AP2-Z functions as a major transcription factor during the early stage of zygote/ookinete development. The other enriched motifs, including the most enriched motif, fully or partially contained the binding motif of AP2-O [TAGC(T/C)A] (Fig 6C) [24]. Consistently, ZUGs contained 190 AP2-O targets, accounting for 37% of ZUGs. Enrichment of the AP2-O binding motif could be due to the large overlap between the target genes of AP2-Z and AP2-O, which is evaluated later in detail. Notably, as with our result for the zygote stage, Witmer et al. reported a similar result for the mature ookinete stage, where the binding motif of AP2-O should be a major *cis*-element for upregulation of genes; in the upstream region of genes whose expression was upregulated more than 8-fold in mature ookinetes compared to female gametocytes, a motif almost identical to the AP2-Z binding motif, G(T/A)GAACA, was detected as the second most enriched motif (next to the binding motif of AP2-O) [29].

### Target genes of AP2-Z are downregulated in ap2-*z*(-)

**Fig 7:**
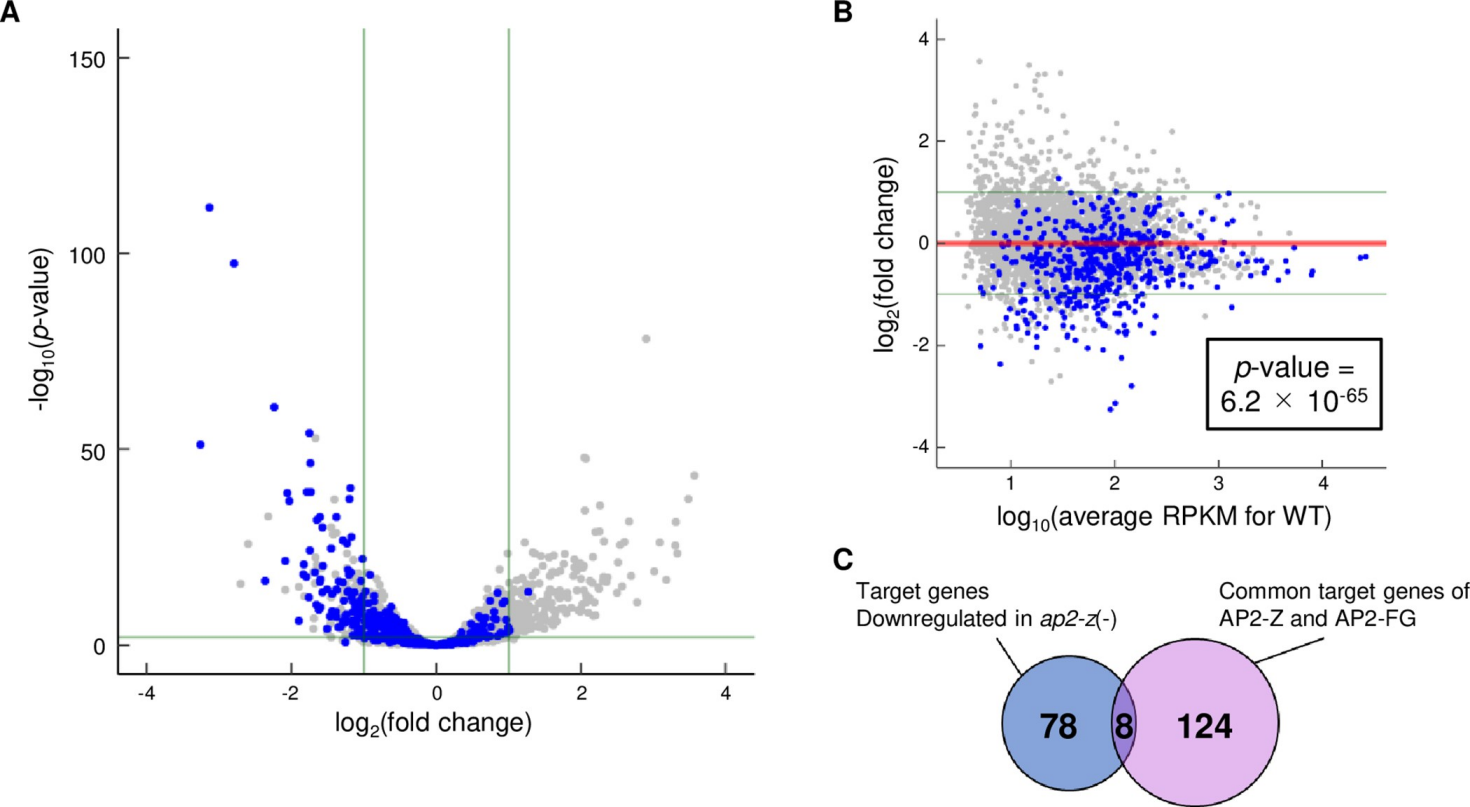
Differential expression analysis between WT and *ap2-z*(-) at 6 hoc. (A) Volcano plot showing differential expression analysis between WT and *ap2-z*(-) at 6 hoc. Blue dots represent the target genes of AP2-Z. Green horizontal line indicates a *p*-value of 0.05. Two vertical lines indicate log_2_(fold change) values of 1 and −1. The plot for *ap2-z* is omitted from the graph [log_2_(fold change) = −4.5, *p*-value adj = 4.9 × 10^−300^]. (B) MA plot showing differential expression analysis between WT and *ap2-z*(-) at 6 hoc. The *p*-value in the box represents a statistically significant difference between the mean values of log_2_(fold change) for AP2-Z targets and the other genes. Two green lines indicate log_2_(fold change) values of 1 and −1, and the red line indicates a log_2_(fold change) of 0. (C) Venn diagram showing the overlap between AP2-Z targets downregulated in *ap2-z*(-) and the common targets of AP2-Z and AP2-FG.

To evaluate the contribution of AP2-Z to transcriptional regulation in zygotes, we performed differential expression analysis between WT and *ap2-z*(-) by RNA-seq. The analysis revealed that 161 genes, excluding *ap2-z*, were significantly downregulated (log_2_(fold change) < -1, *p*-value adj < 0.01) and 184 were significantly upregulated (log_2_(fold change) > 1, *p*-value adj < 0.01) in *ap2-z*(-) compared to WT at 6 hoc (Fig 7A and 7B, S4A and S4B Table). Of these downregulated genes, 86 genes were a target gene of AP2-Z, showing enrichment of the target genes in the downregulated genes (*p*-value = 1.8 × 10^−40^ by Fisher’s exact test). In addition, the mean value of log_2_(fold change) for the target genes was significantly lower than that for the other genes, with a *p*-value of 6.2 × 10^−65^ by Student’s t-test, indicating that the target genes were generally downregulated in *ap2-z*(-) (Fig 7B). Nevertheless, downregulation was only significant for a subset of the target genes (17%). We assumed that downregulation of some targets was not significant because they were already transcribed in female gametocytes, mostly by AP2-FG. Consistently, of 132 genes targeted by both AP2-Z and AP2-FG, only eight were significantly downregulated in *ap2-z*(-) (Fig 7C). In addition, the mean value of log_2_(fold change) for the common targets was significantly lower than that for the other AP2-Z targets, with a *p*-value of 4.8 × 10^−5^ by Student’s t-test.

### Comparative targetome analyses between AP2-FG, AP2-Z, and AP2-O suggests their roles in regulating zygote/ookinete development

The results of this study suggested that gene expression during *Plasmodium* zygote/ookinete development is regulated by AP2-FG, as female-stored mRNA, and AP2-Z and AP2-O, as *de novo* transcripts. In this regard, we attempted to investigate the role of each of these three transcription factors by comparing their target genes.

**Fig 8:**
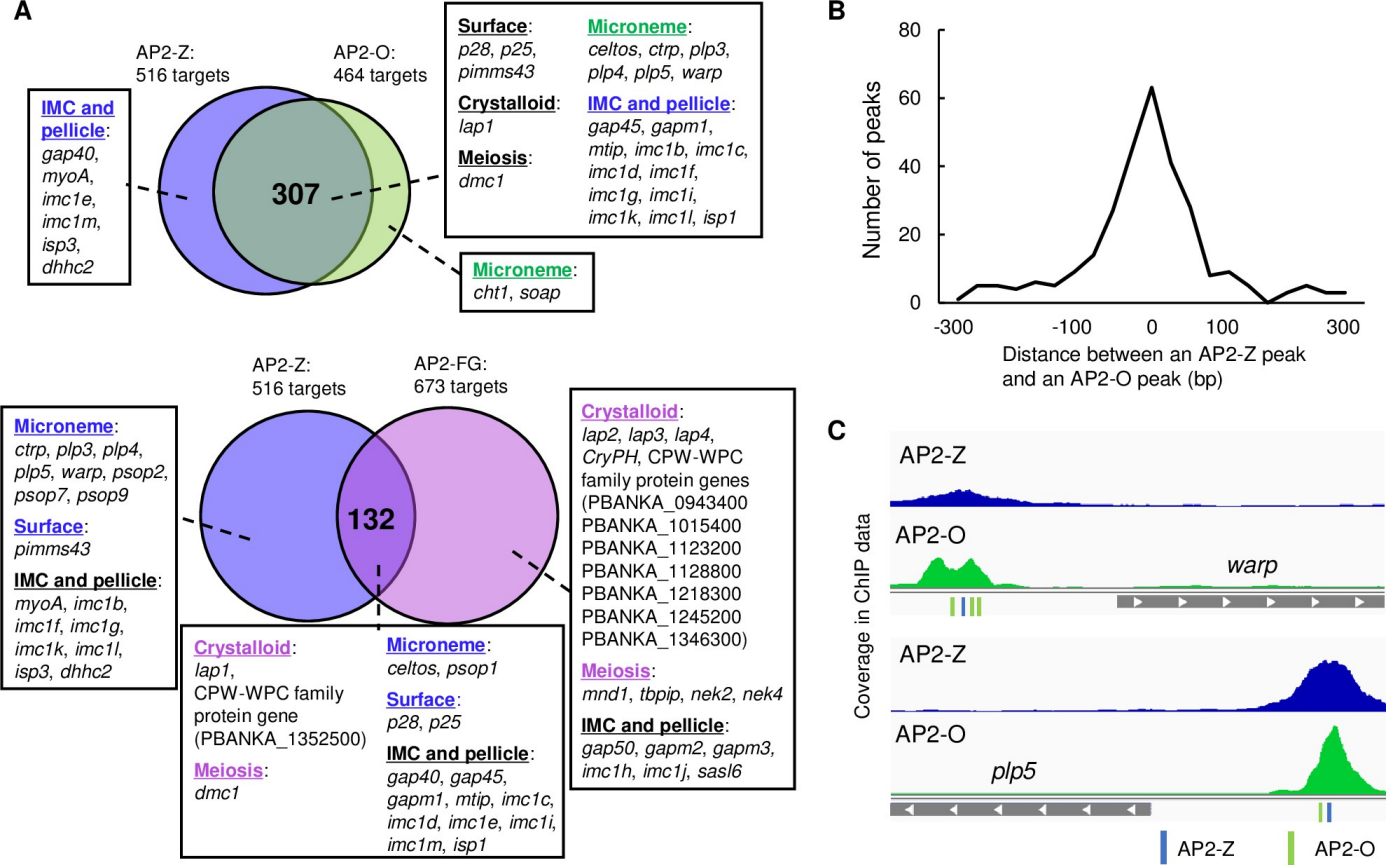
Comparison of the target genes between AP2-Z, AP2-O, and AP2-FG. (A) Venn diagrams showing the overlap between the target genes of AP2-Z and AP2-O (top) and AP2-Z and AP2-FG (bottom). Genes were assigned according to previous literature [24,36–44,50–75]. (B) Distance between ChIP peaks of AP2-Z and AP2-O in the upstream region of their common targets. (C) Representative peak images of the common target genes of AP2-Z and AP2-O. Positions of AP2-Z and AP2-O binding motifs are indicated in blue and green, respectively.

First, we compared the target genes of AP2-Z and AP2-O. The two target gene sets showed a large overlap (307 genes), accounting for 60% of the targets of AP2-Z (Fig 8A, S2 Table). This result was consistent with the above result that the binding motif of AP2-O was enriched in the upstream of ZUGs. These overlapped genes included most of the known microneme genes, ookinete surface protein genes, and IMC/pellicle genes, which are all related to parasite invasion. Therefore, the main role of AP2-Z may be promoting the development of an invasive stage until expression of AP2-O. Accordingly, these common targets are continuously activated by the two transcription factors throughout development from zygote to ookinete, which may be important for efficiently promoting ookinete formation. These common target genes have ChIP binding sites of both AP2-Z and AP2-O in their upstream region. Notably, these binding sites of AP2-Z and AP2-O are located very close to each other in the upstream region of the same genes (mostly within 100 bp). (Fig 8B and 8C). This result suggested that the two transcription factors share the same regulatory regions for their common target genes, and that a major transcriptional regulator gradually switches from AP2-Z to AP2-O in these regulatory regions as zygote/ookinete development progresses.

In contrast, the target genes unique to AP2-Z or AP2-O contained only a small number of known ookinete genes. These ookinete genes in the unique targets contained six known IMC/pellicle genes for AP2-Z and two known microneme genes, *cht1* and *soap*, for AP2-O (Fig 8A, S2 Table). While IMC formation is necessary from the first steps of ookinete formation, *i*.*e*. determination of apical polarity and construction of an apical protrusion [35], microneme is essential for parasite motility and invasion in mature ookinetes. Therefore, these results might suggest that, while regulating the common targets, AP2-Z and AP2-O also regulate their unique target genes in accordance with their own expression pattern during zygote/ookinete development.

Next, we investigated the target genes of AP2-Z and AP2-FG. Since the target genes of AP2-FG contains most of the female-enriched genes [14], we considered that this analysis could possibly provide an indication for the role of female-stored mRNAs unique from that of *de novo* transcripts. The genes common in the two target sets contained 132 genes, representing approximately 25% of the AP2-Z targets (Fig 8A, S2 Table). Several IMC/pellicle genes were included in the target genes of AP2-FG as both common and unique targets (Fig 8A, S2 Table), which, as with previous studies [14,18,19], indicated that female-stored genes are essential for ookinete formation. This is consistent with the fact that fertilized females were able to develop into retort-form ookinetes, even when *ap2-z* was disrupted [10] or fertilized females were treated with a transcriptional inhibitor [19]. On the other hand, the AP2-FG targets contained only two known microneme genes, both of them as common targets, suggesting that although playing a role in the initial steps of ookinete formation, female-stored mRNAs may not largely contribute to the later steps. The other unique targets of AP2-FG contained most of the genes related to meiosis and crystalloid biogenesis (Fig 8A, S2 Table), which are not directly involved in ookinete formation [36–40]. This result suggested that developmental processes other than ookinete formation are mainly regulated by female-stored mRNAs during *Plasmodium* zygote/ookinete development.

### Unique targets of AP2-Z contain an important gene for ookinete development

**Fig 9:**
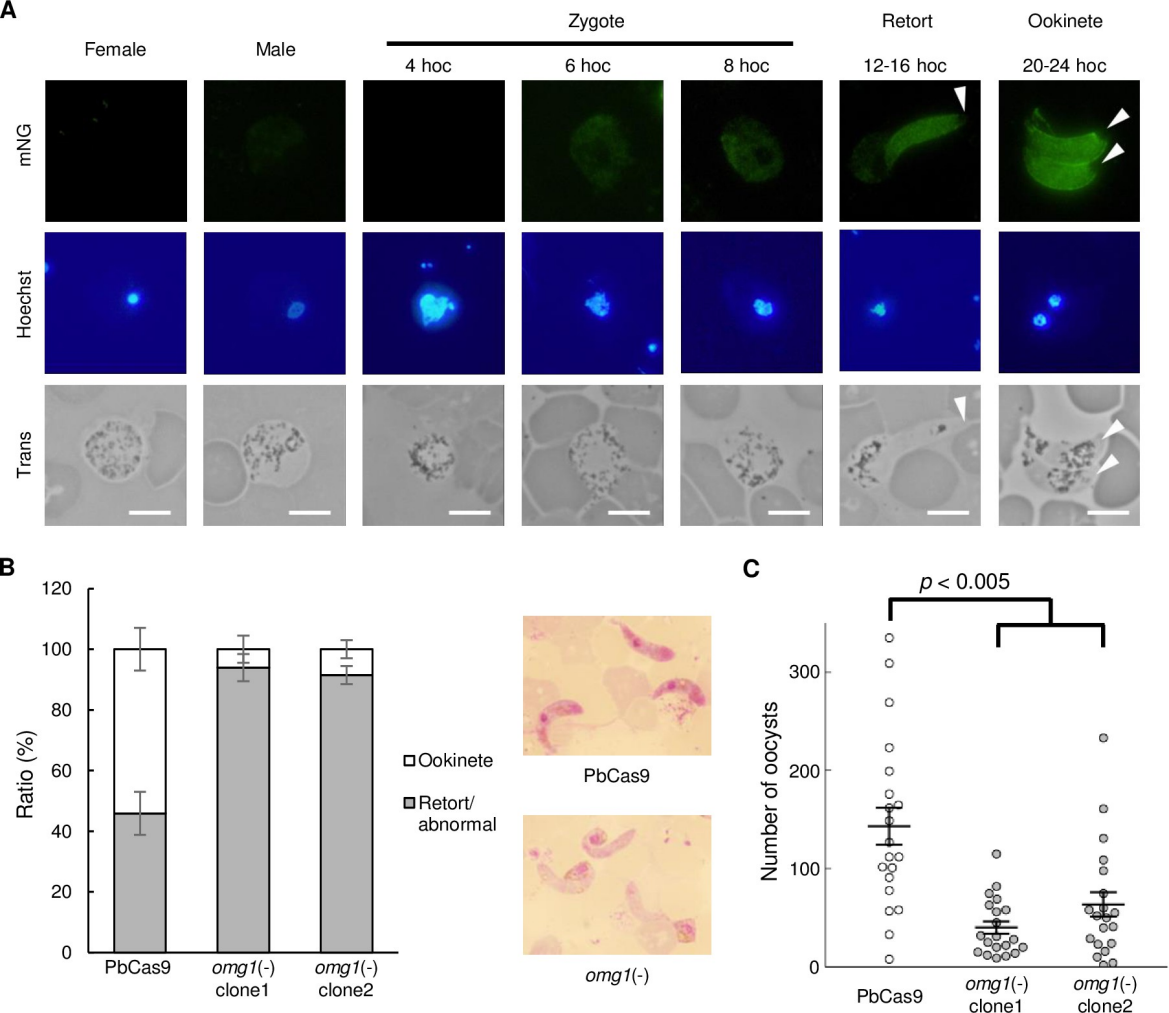
Investigation of one of the unique targets of AP2-Z, *omg1*. (A) Expression of OMG1 in the OMG1::mNG parasite during sexual development. Arrow heads indicate the apical end of the ookinete. Nuclei were stained with Hoechst 33342. Scale bar = 5 μm. (B) Ratio of ookinetes and retort-form/abnormal zygotes to all fertilized cells in PbCas9 and *omg1*(-) at 16 hoc. Right side of the figure shows Giemsa-stained images of ookinetes at 16 hoc in PbCas9 (top) and *omg1*(-) (bottom). Error bars indicate the standard error of the mean (n = 3). (C) Number of midgut oocysts at 14 days post infection for PbCas9 and *omg1*(-). Lines indicate the mean values and the standard error (n = 20). The *p*-value on the graph was calculated by Student’s t-test.

Although the target genes of AP2-Z largely overlapped with those of AP2-O, they also contained a considerable number of unique targets, most of which have not yet been functionally assessed. To evaluate the roles of these unique targets, we assessed one of the unique target genes with an unknown function, PBANKA_0508300 (hereafter named ookinete maturation gene 1 (*omg1*)). Notably, *omg1* was significantly upregulated at 6 hoc compared to gametocytes, with a log_2_(fold change) of 3.33, suggesting its transcription after fertilization. By fusing *mNG* to *omg1* using CRISPR/Cas9 (OMG1::mNG) (S3C Fig), we investigated the *omg1* expression pattern during ookinete development. In the blood stage, both female and male gametocytes of OMG1::mNG showed no fluorescent signal (Fig 9A). After fertilization, a weak signal started to appear at 6 hoc. This signal intensified in retort-form ookinetes and was still observed in matured ookinetes. Fluorescence was observed along the periphery of ookinetes but was absent in the zygote remnant and the apical end (Fig 9A). This pattern of distribution is similar to that of several known IMC components, such as IMC1h and IMC1i [24,41–44]. Therefore, this result suggested that OMG1 is a component of IMC that plays a role in pellicle formation. This was consistent with the comparative targetome analysis between AP2-Z and AP2-O, in which it was suggested that unique targets of AP2-Z include genes required from the first steps of ookinete formation, such as IMC/pellicle genes.

We next disrupted *omg1* by CRISPR/Cas9 [*omg1*(-)] using the PbCas9 parasite, obtaining two clonal lines from two independent transfection experiments (S3D Fig), and investigated its phenotype. Both female and male gametocytes of *omg1*(-) appeared normal and were able to fertilize. In addition, meiosis was also normal in *omg1*(-) as assessed by FACS analysis (S1 Fig). However, ookinete maturation was delayed in *omg1*(-) compared to PbCas9 parasites. In *omg1*(-), the ratio of banana-shaped ookinetes to all fertilized cells was less than 10% at 16 hoc for both clones, whereas more than half of fertilized cells had become banana-shaped ookinetes in PbCas9 at the same time point (Fig 9B). Nonetheless, disruption of *omg1* did not completely impair ookinete development as most fertilized cells became banana-shaped ookinetes prior to 30 hoc.

We further investigated whether the delay in ookinete maturation affects mosquito infectivity. Mice infected with PbCas9 parasites or *omg1*(-) were fed on *Anopheles stephensi* mosquitoes. At 14 days post infection, the number of oocysts in the midgut of infected mosquitoes was significantly reduced in both clones of *omg1*(-) compared to in PbCas9 (Fig 9C). Therefore, because of impaired ookinete development, *omg1*(-) might delay in penetrating the peritrophic membrane to reach the basal lamina and hence be exposed to the hostile environment of the mosquito midgut for a longer time. Collectively, these results suggested that the unique targets of AP2-Z contain genes that are important for ookinete maturation.

## Discussion

Previous studies showed that, in *P*. *berghei*, female-stored mRNA is essential for zygote/ookinete development; thus, disruption of the DOZI complex completely abolishes meiosis and the ookinete conversion of fertilized females [16,18]. Moreover, fertilized females are able to develop until the retort stage of ookinetes in the presence of a transcription inhibitor, which suggested that female-stored mRNAs are sufficient for promoting the initial step of ookinete conversion [19]. On the other hand, our previous studies revealed the essential role of *de novo* transcription by AP2-O in ookinete maturation, where disruption of *ap2-o* resulted in developmental arrest at the retort stage of ookinetes [23]. Putting these studies together suggests that *Plasmodium* zygote/ookinete development is promoted by female-stored mRNA until the retort stage of ookinetes and *de novo* transcription by AP2-O in the later stage. Accordingly, transcriptional activity in zygotes has been considered quiescent, similar to animal embryos that are transcriptionally silent in the early stage after fertilization [21,22]. In this study, by investigating the roles of a novel AP2-family transcription factor, AP2-Z, we demonstrated that in *P*. *berghei*, *de novo* transcription after fertilization is actually activated by AP2-Z before formation of apical protrusion and is essential for zygote/ookinete development. Therefore, our results indicated that female-stored mRNAs and *de novo* transcripts by AP2-Z both promote the zygote development.

**Fig 10:**
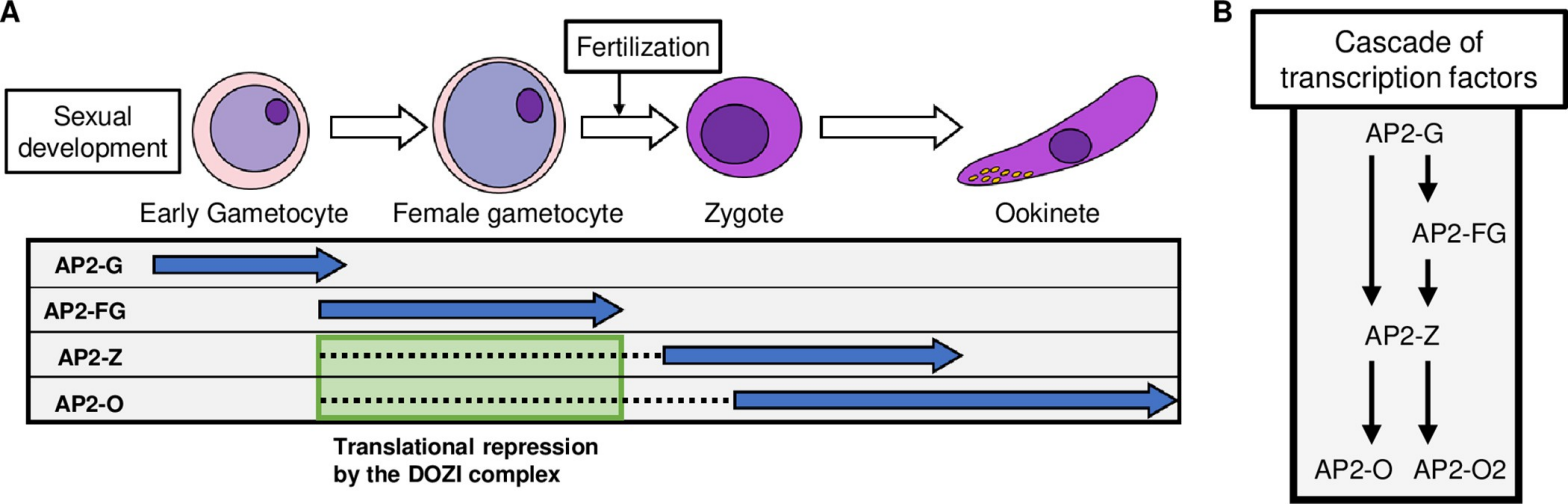
Cascade of transcription factors during the sexual development of *Plasmodium*. (A) Schematic illustration of the sequential expression of sexual transcription factors: AP2-G, AP2-FG, AP2-Z, and AP2-O. Blue arrows indicate a period of protein expression and dashed lines indicate a period of translational repression. (B) Schematic illustration showing the hierarchical relationship of sexual transcription factors during *Plasmodium* sexual development.

In *Plasmodium*, a single DNA-binding transcription factor, such as AP2-G and AP2-O, comprehensively regulates certain stage-specific genes [10,14,24,45,46]. Such a simple mechanism of gene activation might enable regulation of the entire *Plasmodium* life cycle by only a small number of sequence-specific transcription factors (approximately 30 AP2-family transcription factors and a few others have been identified to date [47–49]). If the life cycle is simply proceeded by sequential expression of such master transcription factors, it is plausible to hypothesize that they make up a large cascade covering the entire life cycle; i.e., every stage-specific transcription factor, while regulating the respective stage-specific genes, activates the transcription factors responsible for regulation of the next stage. In this study, we confirmed such a cascade during sexual development via the promoter assay of *ap2-z*; that is, transcription of *ap2-z* in female gametocytes was completely depleted upon disruption of the binding motifs of AP2-G and AP2-FG in the upstream region. Furthermore, through target analysis of AP2-Z, we revealed that AP2-Z targets *ap2-o* and *ap2-o2*, which are essential transcription factors for ookinete maturation. These results indicated that a dynamic cascade of transcription factors that starts from AP2-G occurs during the entire process of *Plasmodium* sexual development (Fig 10A and 10B). If such a simple cascade of transcription factors was found throughout the entire life cycle of *Plasmodium*, we should always be able to identify transcriptional regulators for the next developmental stage from target analysis of a certain transcriptional activator, just as we found *ap2-z* from the target genes of AP2-G and AP2-FG in this study.

In conclusion, this study revealed the essential role of transcription by AP2-Z in zygotes and a dynamic cascade of transcription factors during *Plasmodium* sexual development. Moreover, our comparative targetome analyses provided new insights into the mechanisms regulating zygote/ookinete development. To further expand our knowledge of the molecular mechanisms underlying zygote/ookinete development and transmission from vertebrate hosts to mosquitoes, it is important to evaluate the functions of individual target genes. Especially, we believe that investigation of the unique target genes of AP2-Z could help us elucidate new aspects of the mechanism regulating ookinete development because these genes are not readily identified in transcriptome analyses of gametocytes or ookinetes. In addition, although most of the unique target genes of AP2-Z have not yet been functionally investigated, they may well be important for zygote/ookinete development, as shown in this study with *omg1*.

## Materials and methods

### Ethics statement

All experiments in this study were performed according to recommendations in the Guide for the Care and Use of Laboratory Animals of the National Institutes of Health in order to minimize animal suffering. All protocols were approved by the Animal Research Ethics Committee of Mie University (permit number 23–29).

### Parasite preparation

All parasites used in this study were inoculated in Balb/c or ddY mice. The *ap2-z*(-) and DOZI::GFP parasites were derived from the WT ANKA strain, and all other transgenic parasites were generated by the CRISPR/Cas9 system using Cas9-expressing parasites called PbCas9 [31]. Ookinete cultures were performed as follows. First, infected blood was intraperitoneally injected into phenylhydrazine (nacalai tesque)-treated mice. When the parasitemia reached approximately 3%, the mice were fed sulfadiazine (Sigma) in their drinking water (10 μg/ml) to kill asexual stage parasites. After two days of sulfadiazine treatment, infected blood was withdrawn from the mice and diluted in culture medium (RPMI1640 medium whose pH was adjusted to 8.0 with NaOH solution, supplemented with fetal calf serum and penicillin/streptomycin, achieving final concentrations of 20% and 1%, respectively). Blood samples were then passed through a column filled with cellulose powder CF11 or a Plasmodipur filter for ChIP-seq and RNA-seq experiments, and the parasites were incubated at 20°C. Midgut oocysts and sporozoites were counted from the midgut of infected mosquitoes at 14 days post-infective blood meal.

### Generation of transgenic parasites

For tagging DOZI with GFP, the conventional homologous recombination method was used as previously reported [23]. Briefly, two homologous regions were cloned into the *gfp*-fusion vector to fuse *dozi* in frame with *gfp*. The vector was linearized by restriction enzymes and transfected into parasites by electroporation. Mutants were selected with 70 μg/mL pyrimethamine in drinking water. The previously reported CRISPR/Cas9 system was used to generate transgenic parasites by CRISPR/Cas9 [31]. Briefly, PbCas9 parasites, which constitutively express Cas9 endonuclease, were transfected with donor DNA prepared by the overlap PCR method and sgRNA vector by electroporation. After transfection, mice infected with the transfectants were treated with 70 μg/mL of pyrimethamine (Sigma) in their drinking water for three days to select the desired mutants. All clonal parasites were obtained by limiting dilution methods, and genotyping was performed by PCR and Sanger sequencing. All primers used in this study are listed in S5 Table (No. 1–39).

### Fluorescence-activated cell sorting analysis

FACS analysis was performed using the LSR Fortessa (Becton Dickinson). Nuclei were stained with Hoechst 33342. Sulfadiazine-treated parasites, before starting ookinete cultures or at 8 hoc, were gated with forward-scatter and Hoechst fluorescence intensity. Gated cells were assessed for Hoechst fluorescence intensity (450/50).

### RNA immunoprecipitation experiments

For the RIP experiments, mice infected with DOZI::GFP parasites were treated with sulfadiazine in their drinking water to kill asexual parasites. After two days of drug treatment, whole blood was withdrawn from the infected mice and washed once with RPMI medium. After lysing red blood cells in ice-cold lysis solution (1.5 M NH4Cl, 0.1 M KHCO_3_, 10 mM EDTA), the samples were subjected to RIP experiments, which were performed using RiboCluster Profiler (MBL Co., Ltd.) according to the manufacturer’s instructions. Briefly, cells were lysed in the provided lysis buffer, and the lysate was mixed with antibody-free Protein A agarose beads then incubated at 4°C as a preclear step. Next, the precleared cell lysate was separated from the beads by centrifugation. A small portion of the precleared sample was collected for input RNA quantification, and the rest was mixed with antibody-immobilized Protein A agarose beads and incubated at 4°C. During this process, anti-GFP polyclonal antibody (Abcam, ab290) and normal rabbit IgG supplied in the kit were used for immunoprecipitation of DOZI-associated RNAs and as a negative control, respectively. Finally, RNA was purified from the beads and subjected to RT-qPCR analysis. Three biologically independent samples were prepared and used for the analysis.

### RT-qPCR analysis

cDNA was synthesized from total RNA or immunoprecipitated RNA using PrimeScript RT reagent Kit with gDNA Eraser (Takara). RT-qPCR analysis was performed using TB Green Fast qPCR Mix (Takara) and Thermal Cycler Dice Real Time System II (Takara). All primers used in this study are listed in S5 Table (No. 40–45).

### ChIP-seq and sequencing data analysis

The ChIP-seq experiments were performed as described previously [24]. Briefly, AP2-Z::GFP parasites at 6 hoc were fixed by adding formalin solution to achieve the final concentration of 1% and incubating the solution at 30°C. After fixing, red blood cells were lysed in ice-cold 1.5 M NH4Cl solution. Next, the residual cells were lysed in SDS lysis buffer, and the lysate was sonicated using Bioruptor (Cosmo Bio) to shear chromatin. A small portion of the sonicated lysate was collected as input samples. ChIP samples were immunoprecipitated from the sonicated lysate with anti-GFP polyclonal antibodies (Abcam, ab290) immobilized on Dynabeads Protein A (Invitrogen). DNA fragments were purified from the ChIP and input samples, and then used for library construction, which was performed with a KAPA HyperPrep Kit according to the manufacturer’s instructions. The library was then sequenced by Illumina NextSeq. Two biologically independent experiments were performed.

The obtained sequence data were mapped onto the reference genome sequence of *P*. *berghei* ANKA, downloaded from PlasmoDB 46, by Bowtie2. Reads that aligned more than two times were removed from the mapping data. Using the mapping data from the ChIP and input samples, peaks were identified using the macs2 callpeak function with fold enrichment > 4.0 and *q*-value < 0.01, and common peaks between the two experiments were used for further analysis. Binding motifs were predicted by analyzing enrichment of motifs within 50 bp from peak summits using Fisher’s exact test (the method was previously described in detail [24]). Genes that had peaks within the region upstream of 1,200 bp from ATG were identified as target genes. Parameters for all programs were set to the default, unless specified otherwise.

### RNA-seq and sequence data analysis

Total RNA was extracted from Plasmodipur-filtered mouse blood infected with WT or *ap2-z*(-) using the Isogen II reagent (Nippon gene). Briefly, red blood cells were lysed in ice-cold 1.5 M NH_4_Cl solution. The residual cells were then lysed in Isogen II, and total RNA was purified from the lysate according to the manufacturer’s instructions. From the total RNA, RNA-seq libraries were prepared using the KAPA mRNA HyperPrep Kit and sequenced by Illumina NextSeq. Three biologically independent experiments were performed for each sample. The obtained sequence data were mapped onto the reference genome sequence of *P*. *berghei* by HISAT2, setting the maximum intron length threshold to 1,000. The mapping data for each sample were analyzed by featureCounts and compared using DESeq2. The reads per kilobase of transcript per million mapped reads for each gene were calculated from the featureCounts results; genes with values of less than five in all three datasets for WT at 6 hoc were removed from the differential expression analysis. Genes in subtelomeric regions were also removed. For all programs, the parameters were set to the default, unless specified otherwise.

## Supporting information

S1 Fig FACS analysis of Hoechst signal. From the top, WT before starting ookinete culture, WT at 8 hoc, *ap2-z*(-) at 8 hoc and *omg1*(-) at 8 hoc were shown. Nuclei were stained with Hoechst 33342.
(TIF)

S2 Fig Alignment of amino acid sequences of a putative AP2 domain of AP2-Z for *Plasmodium* species. Positions at which all sequences have an identical amino acid are indicated by two asterisks, whereas positions with amino acid residues of the same property are indicated by one asterisk. Amino acid sequences were retrieved from PlasmoDB.
(TIF)

S3 Fig Genotyping of transgenic parasites developed in this study. (A) AP2-Z::GFP. (B) *ap2-z*(-). (C) OMG1::mNG. (D) *omg1*(-).
(TIF)

S1 Table List of peaks identified in the ChIP-seq experiments. (A) Experiment 1. (B) Experiment 2.
(XLSX)

S2 Table List of target genes of AP2-Z. (XLSX)

S3 Table List of zygote-upregulated genes. (XLSX)

S4 Table List of differentially expressed genes in *ap2-z*(-). (A) Significantly downregulated genes. (B) Significantly upregulated genes.
(XLSX)

S5 Table List of primers used in this study. (XLSX)
