# Supplementary figures and images for: Identification of a novel AP2 transcription factor in zygotes with an essential role in Plasmodium ookinete development
Source: PLoS Pathog. 2022 Aug 10;18(8):e1010510. doi: 10.1371/journal.ppat.1010510 (PMC9394825; doi:10.1371/journal.ppat.1010510)

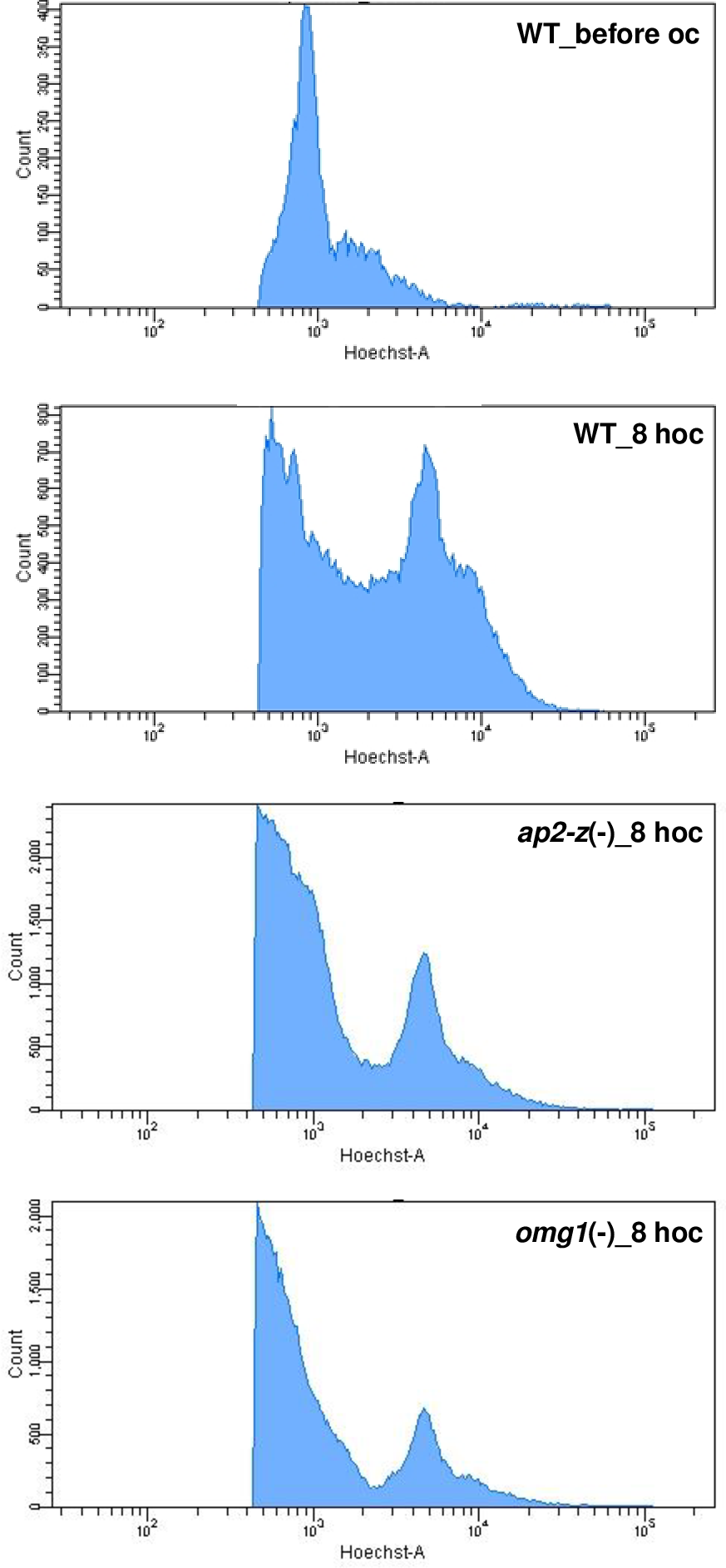

Supplement: S1 Fig — From the top, WT before starting ookinete culture, WT at 8 hoc, ap2-z(-) at 8 hoc and omg1(-) at 8 hoc were shown. Nuclei were stained with Hoechst 33342. (TIF) [file ppat.1010510.s001.tif]

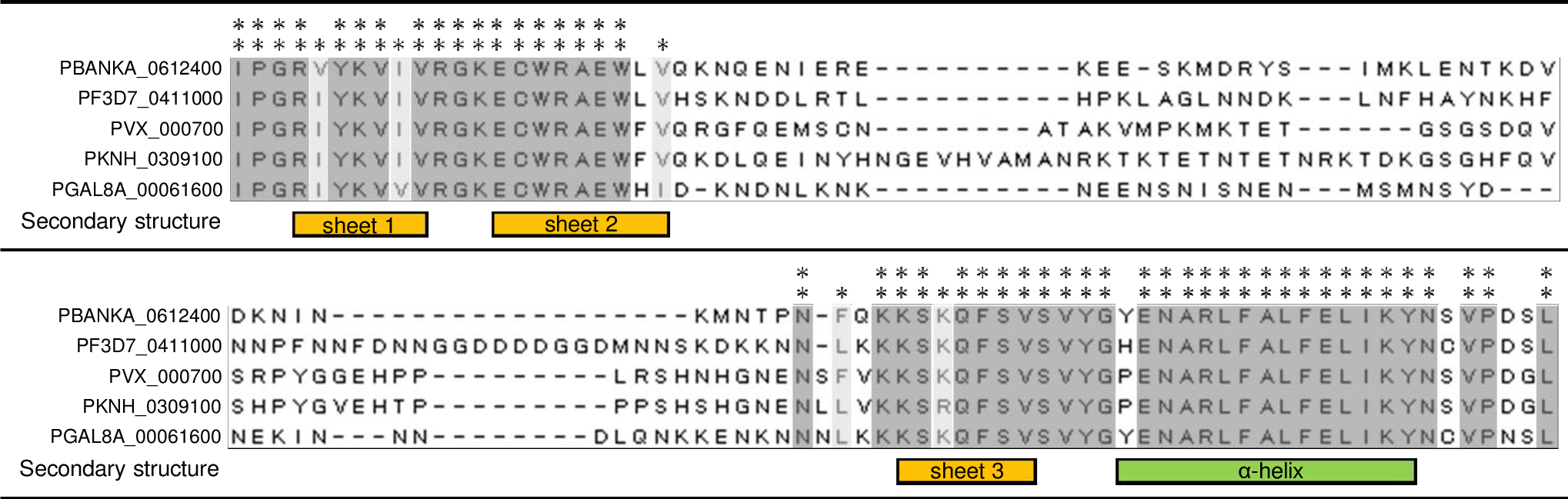

Supplement: S2 Fig — Positions at which all sequences have an identical amino acid are indicated by two asterisks, whereas positions with amino acid residues of the same property are indicated by one asterisk. Amino acid sequences were retrieved from PlasmoDB. (TIF) [file ppat.1010510.s002.tif]

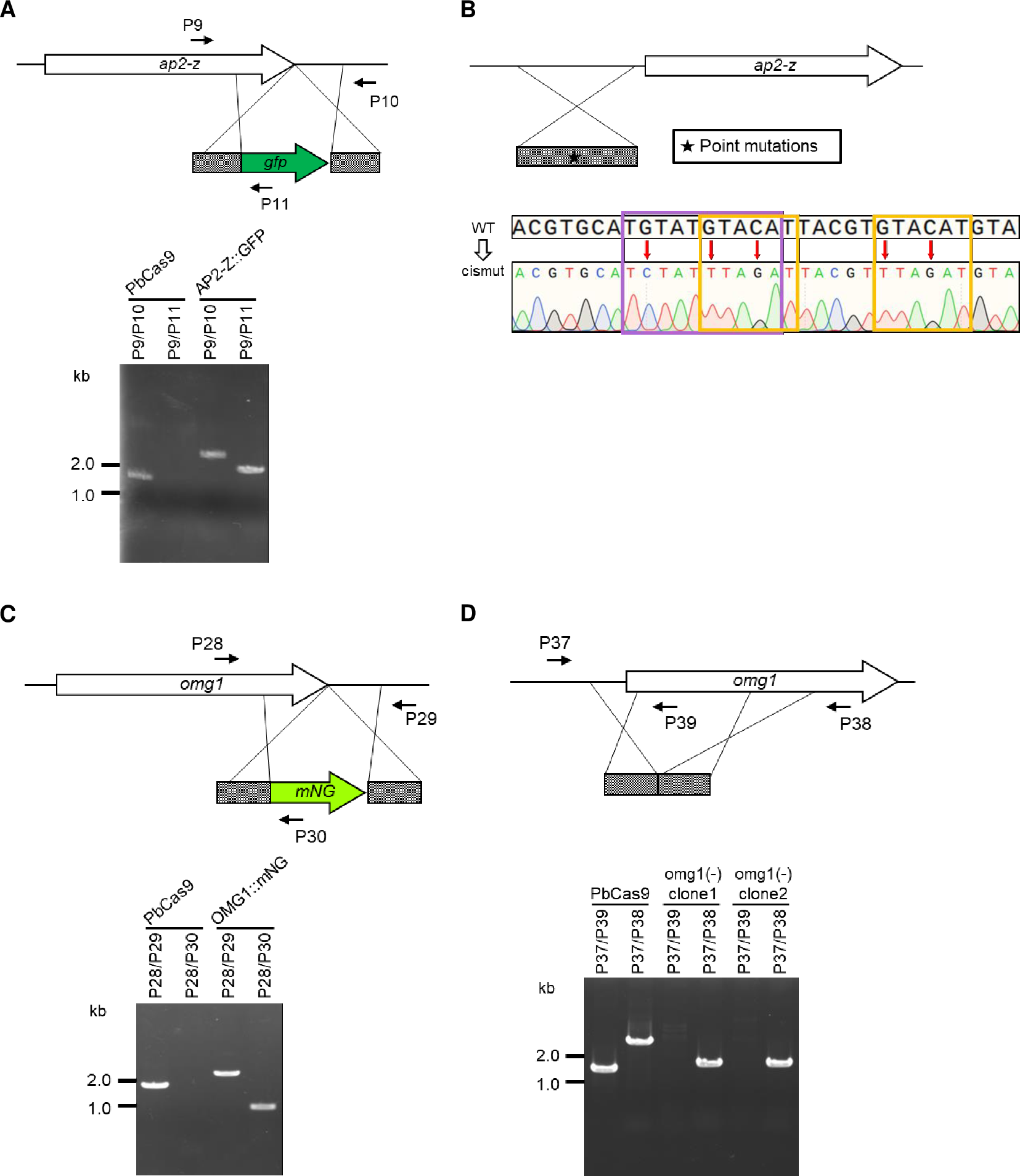

Supplement: S3 Fig — (A) AP2-Z::GFP. (B) ap2-z(-). (C) OMG1::mNG. (D) omg1(-). (TIF) [file ppat.1010510.s003.tif]
